# Biographical Feature: Joseph E. McDade, PhD

**DOI:** 10.1128/jcm.00648-26

**Published:** 2026-07-20

**Authors:** Erik Munson

**Affiliations:** 1 Center for History of Microbiology and ASM Archives (CHOMA), Baltimore, Maryland, USA; 2 Wisconsin Clinical Laboratory Network Laboratory Technical Advisory Group, Madison, Wisconsin, USA; Vanderbilt University Medical Center, Nashville, Tennessee, USA

## TEXT

**Figure:**
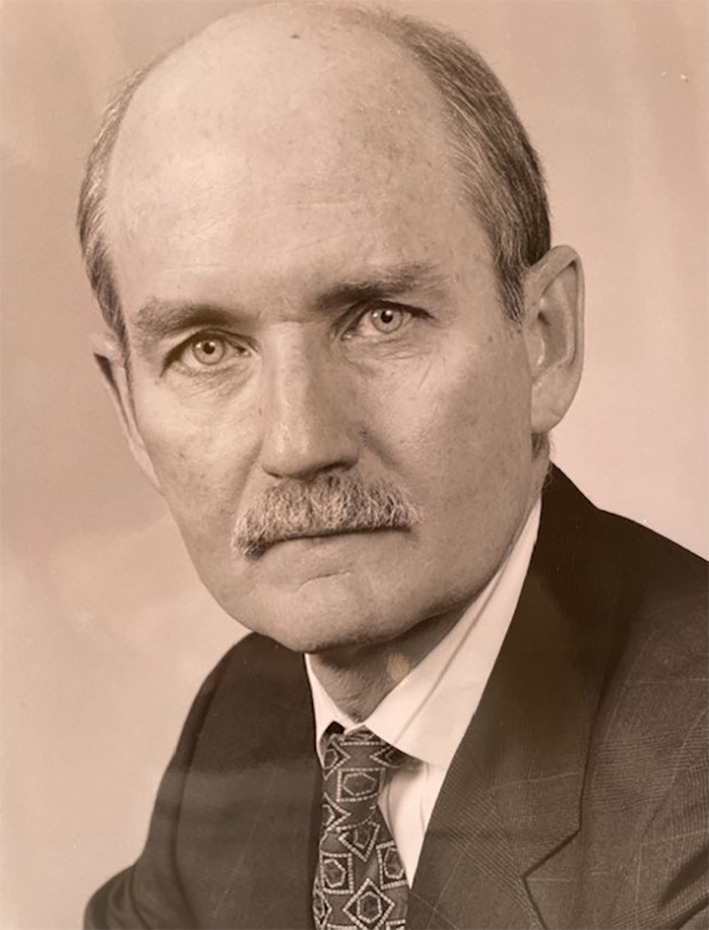

“At this initial stage of the investigation, the agent…has been distinguished by the following properties: the characteristic disease that it produces in guinea pigs; the characteristic death pattern of chick embryos after yolk-sac inoculation; morphology of the organism in smears of infected yolk sacs and guinea-pig peritoneal exudates; gram-negative characteristics; failure to grow on ordinary bacteriologic media, such as trypticase soy agar, blood agar, and thioglycollate broth; and specific immunofluorescent staining with convalescent-phase serums of patients with … disease. As mentioned above, the organism has since been cultivated on supplemented Mueller-Hinton medium, and studies of its classification are in progress (1). We cannot say as yet whether the organism has previously been isolated from other sources, but it seems clear that this is the first demonstration of its role in human disease. The properties listed above differentiate it from other known microbes causing human disease” (2).

On the 50^th^ anniversary of the pragmatic thought processes and traditional laboratory techniques that were meticulously devoted to the ultimate characterization of a Gram-negative bacterium as etiology of an historic outbreak-related lower respiratory tract disease, it is more than apropos to briefly chronicle the career of Dr. Joseph E. McDade, a rickettsiologist at the Centers for Disease Control and Prevention (CDC), who contributed largely to these findings. This story may be viewed as being a bit atypical to the eyes of some in that a programmed and orchestrated career ascension, with progressive accomplishments, was not clearly evident for this distinguished microbiologist. With great humility and an attitude of service, Dr. McDade sprinkled anecdotes such as “some of the events along the way seemed miraculous,” “it just makes you wonder, though, how things turned out,” and “(with a chuckle) circumstances when I was pulled from the abyss” into a frank discussion that reflected on his storied career.

Similar to CDC microbiologist Dr. Clyde Thornsberry, who was the subject of a previous *Journal of Clinical Microbiology* biographical feature (3), Dr. McDade’s story begins in the vicinity of the United States Appalachian Mountains. Dr. McDade was raised in Cumberland, Maryland. He recalled one of his grandfathers being a coal miner, as well as a bootlegger who doubled as a saloon owner. His father worked for the railroad, and his mother was a strong influence in his early life. Like many, Dr. McDade toiled through some of his high school years without having specific vocational aspirations. He contemplated becoming an English teacher, as he was fond of elocution. Elocution was most certainly pervasive as he described what happened next, “I was literally sitting on my front steps in April of the year that I graduated from high school and up pulled my uncle (married without children) in a car with tobacco juice all down the driver’s side. He said, ‘Come on, let’s go for a ride.’” “We drove all over, and he asked me what I was going to do.” When the thought of becoming an English educator was offered, the uncle countered, “Oh, god, you are going to starve to death. You ought to be a doctor like one of my classmates from high school.” Dr. McDade related that the family member agreed to pay his college tuition if he agreed to enroll as a pre-medicine student. Dr. McDade finished, “I didn’t really have any strong feeling one way or the other, so he literally gave me some money right on the spot.”

This subsidy lasted through 3 years of undergraduate study at Western Maryland College (now known as McDaniel College) in Westminster, Maryland, before the generous relative was beset with financial difficulties. Dr. McDade was able to cover the expenses of his seventh semester through summer jobs and an unexpected check from a neighbor that covered the shortfall, but was faced with the possibility of not being able to complete his senior year. He then waxed, “I was home for Christmas break (late in 1961), feeling sorry for myself. It was New Year’s Eve, and I can still see myself sitting in the dining room by myself with a half-empty bottle of cheap red wine, and I was feeling absolutely awful. So, I sat down and started writing a letter, ‘Dear President Kennedy…’ that lamented how the college was unable to lend me the funds to finish my schooling and dropped it in the mailbox.” (Author’s note: Dr. McDade did confess to having a hangover [and regret] the next day.) Upon return to the college for the spring semester, Dr. McDade was summoned to the registrar’s office because the school received a telephone call directly from the White House. A student loan was procured, accepted, and eventually repaid.

The generous uncle was unfortunately unable to continue his funding of Dr. McDade’s medical school aspirations. Therefore, Dr. McDade turned to the graduate school application. Not surprisingly, another entertaining anecdote followed, “I had applied for a National Defense Education Act fellowship. Since I was graduating and didn’t know what was in my future, I and a few classmates inquired about local lab jobs (FDA, NIH, and others). Later on, I was playing Bridge down in the student center near our mailboxes, and a friend showed me a (rejection) letter on typical government letterhead. So, I go pick up my mail, and a similar letterhead was on mine; I walked back to the Bridge table and threw mine in the trash can. A little while later, someone in the group dumped a full ashtray down into the trash can. I wasn’t paying any attention, but evidently one of the letters was slightly lit because it started to smolder. I picked it up. It was my letter, and I opened it, ‘Dear Mr. McDade, You have been awarded a National Defense Education Act Fellowship at the University of Delaware graduate school. If we don’t hear from you in 60 days, we will assume that you don’t want it.’ I saved that tinged letter for a long time…through several moves. I lost it somewhere along the line; I wish that I still had it because that’s when I realized that someone was looking out for me.”

Dr. McDade recalled that scientific interests began to drift toward microbiology in his senior year of college while alluding to an overall science renaissance in college (including 1 year as a teaching assistant for introductory biology laboratory courses), as his exposure to science courses was not as strong during his high school education. He earned a master’s (1965; biology) and doctoral (1967; microbiology) degree at the University of Delaware in Newark, Delaware. Early in his graduate school tenure, Dr. McDade had the opportunity to work with Dr. William R. A. Bailey (of *Bailey & Scott’s Diagnostic Microbiology* fame—Dr. McDade quipped that “he was on his second edition at the time, which was only about a half-inch thick”) and co-authored a paper on *Escherichia coli* antigenic characterization (4). (A past *Journal of Clinical Microbiology* biographical feature (5) makes mention of Dr. Richard B. [Tom] Thomson having a Delaware-based professional association with the second author of that classic pedagogical text.) Doctoral studies were conducted under the tutelage of Dr. Marenes R. Tripp, a parasitologist by trade who had an additional interest in oyster immunology (6, 7).

As an undergraduate, Dr. McDade was heavily involved in United States Army Reserve Officers’ Training Corps activities (including earning the title of Battalion Commander during the second semester of his senior year). As a result, Dr. McDade had a military obligation following completion of doctoral studies. From 1967 to 1969, he was assigned, with the rank of captain, to the United States Army Biological Laboratories in Fort Detrick, Maryland, as a research microbiologist. He worked primarily in the virology division, where scientists also undertook investigations in rickettsiology. “The rickettsial field was very much low supply, low demand at that time. There weren’t many of us, and there weren’t many opportunities.” However, in yet another circumstance that was termed “fortuitous,” Dr. McDade was granted the opportunity to attend a rickettsiology conference at the Walter Reed Army Medical Center outside of Washington, D.C., that was arranged by the United States Army Commission on Rickettsial Diseases. He presented on a novel plaque enumeration assay specific to *Rickettsia* spp. (8), was introduced to ranking military officers of all branches and civilians (including researchers from the University of Maryland Medical School) with obvious interests in rickettsiology, and, furthermore, met a young United States Army major and veterinarian named David Huxsoll. Dr. Huxsoll presented on a fatal hemorrhagic syndrome in United States military German shepherd dogs stationed in Vietnam called tropical canine pancytopenia (9). Photomicrographs of intracytoplasmic inclusions consistent with rickettsial bacteria that were found in mononuclear cells of circulating blood and canine organ impressions were shared by Major Huxsoll; the eventual cultivable disease etiology was *Ehrlichia canis* (10).

One ranking member in attendance at that conference later applied for a grant on behalf of the Office of Naval Research to investigate reports of extra-human reservoirs of epidemic (i.e., louse-borne) typhus. Dr. McDade recollected, “they were looking for somebody who would go overseas and be their person on the ground and do some work, and there were very few people that they could ask about. They came across my name probably because they remembered me from being at that conference.” This netted Dr. McDade two secondings to United States Naval Medical Research Units 3 and 5 (Cairo, Egypt (11) and Addis Ababa, Ethiopia (12), respectively) between 1971 and 1975. Interestingly, data supporting those initial reports of epidemic typhus having an extra-human reservoir actually represented a laboratory artifact. At around that time, the CDC was interested in adding expertise to its rickettsiology division due to an unexpected and unexplained increase in rickettsial diseases in the United States. The now-CDC employee Dr. McDade and an entomologist co-worker initiated annual surveillance of tick vectors for *Rickettsia* spp. in 1975 and 1976. That work was subsequently placed on the back burner due to an important reassignment within the CDC for Dr. McDade.

**Fig 1:**
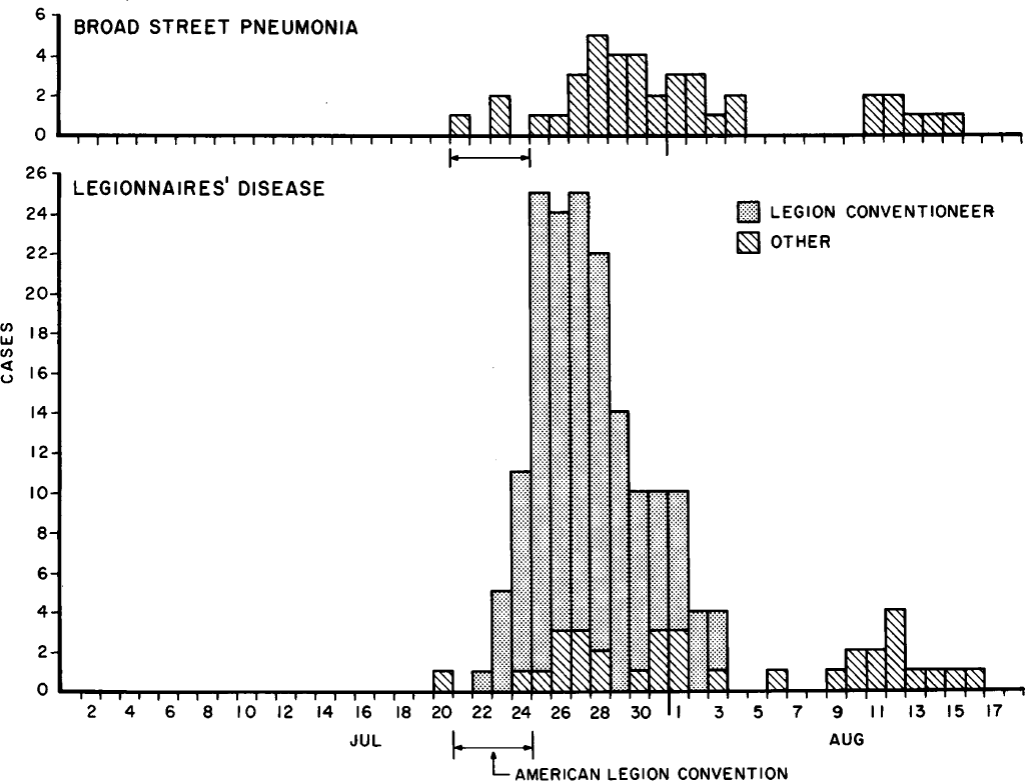
Dates of onset of illness in cases of Legionnaires’ disease and cases of Broad Street pneumonia in Philadelphia, July 1–August 18, 1976. Dates of onset of two cases of Legionnaires’ disease are unknown. Reprinted with permission from Fraser et al (13). ©1977 Massachusetts Medical Society. License Number: 6281380230456.

Fraser et al. (13) reported on 182 cases of pneumonia (29 of which were fatal) that primarily were noted among individuals attending an American Legion convention in Philadelphia, Pennsylvania, in July of 1976. Definitions of Legionnaires’ disease and Broad Street pneumonia (Fig. 1) were provided within this publication. Satisfaction of both clinical and epidemiologic criteria was necessary for assignment as Legionnaires’ disease. Clinical criteria required disease onset between July 1, 1976, and August 18, 1976, and illness characterized by cough and fever (or fever and chest radiology consistent with pneumonia). Epidemiologic criteria either required attendance at the American Legion Convention in Philadelphia from 21 to 24 July 1976 or entrance into an implicated hotel between July 1, 1976, and the onset of illness. Alternatively, individuals were diagnosed with Broad Street pneumonia when clinical criteria were satisfied, but the patient had only set foot within one block of the hotel (rather than enter the hotel or attend the conference) between July 1, 1976, and the onset of illness. Eight epidemiologic surveys were devised by experts and discussed in the publication. These included two case-control surveys, a hotel guest survey, a hotel employee survey, a hospital survey, a roommate survey, and an all-attendee survey. Initial findings revealed a common-source outbreak from an airborne etiology. Exposure was suspected at the entrance to the hotel and in areas immediately surrounding the hotel. Employees were further determined to exhibit some degree of immunity.

When reflecting on his important laboratory contributions toward elucidation of the 1976 respiratory outbreak, the newly employed Dr. McDade commented, “The Legionnaires’ disease work was entirely unexpected. The only reason that I got involved in it was the extremely remote possibility that it was pneumonia caused by Q fever rickettsia…what they were trying to do was rule out all possibilities. We would get some post-mortem patient tissues and see if you could find any rickettsia, specifically Q fever rickettsia. But even before that, they would send around serum specimens from various patients and you would test those against the existing supply of antigens that you have in your lab, and I got ‘zero, zero, zero, zero.’”

Later attempts to cultivate the Q fever rickettsial agent involved intraperitoneal inoculation of experimental guinea pigs with patient tissue homogenates, with subsequent assessment for fever in the animal. Significant findings observed at that juncture would reflex to protocols that dated back upwards of five decades—inoculation of embryonated eggs with guinea pig organ emulsions. Dr. McDade wrote, “these protocols to isolate rickettsiae were designed to eliminate any contaminating bacteria that might be present in the clinical specimen, which could vary considerably depending on the source and condition of the specimen.” Of additional importance, penicillin and streptomycin were added to emulsions that were placed into eggs because these agents were not deleterious to *Rickettsia* spp. recovery and would suppress the growth of any bacterial contaminants. Yolk sacs from eggs that died following at least 3 days of incubation were examined for microbes by Giménez staining (14). On occasion, rare bacteria were observed in these yolk-sac cultures but were initially dismissed as contaminants due to the high unlikelihood that the Pennsylvania outbreak of respiratory illness would be caused by an obligate intracellular bacterium. Dr. McDade later opined, “bacteria that cause pneumonia in humans were first isolated in the 19th century and there was no particular reason to suspect that a novel bacterium would emerge after such a lengthy interval.”

Some time later, a conversation at a local holiday gathering (“pig-in-the-blanket in one hand and a glass of red wine in the other”) served as a pseudo-“call to arms.” Again, this encounter is best related by Dr. McDade, “I ran into this guy who walked up to me (after being told that I work at the CDC). So, I guess that after the guy had a drink or two, he felt emboldened to come over and vent to me a little bit. He said, ‘I know you scientist types are weird. You have beards, long hair, and all of that. But we *count* on you guys to do this kind of thing. I just don’t know why you haven’t been able to figure this thing out.’ I have to tell you, he hit a nerve.” During some end-of-the-year tidying of his laboratory bench, Dr. McDade encountered some stained slides of yolk-sac cultures. “I’ll go back and take a look at them again. And I’m looking, I’m looking, and I’m looking and all of a sudden WHAM I come across a couple of cells that were absolutely loaded with bacteria.” Dr. McDade then decided to return to original (frozen) guinea pig emulsions, then re-inoculate embryonated eggs—this time without penicillin and streptomycin treatment.

**Fig 2:**
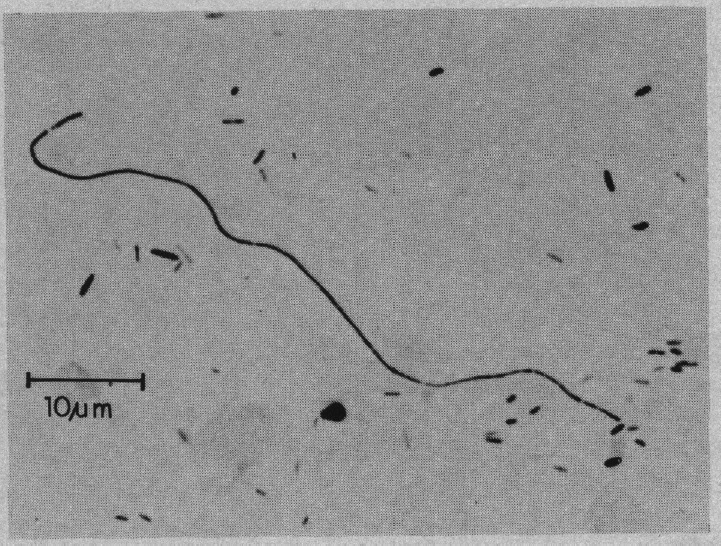
Photomicrograph of the bacteria of Legionnaires’ disease in yolk sac stained by the Giménez method. Reprinted with permission from McDade et al (2). ©1977 Massachusetts Medical Society. License Number: 6281380124112.

Growth of the unknown etiology in this set of experiments was far more florid and abundant (Fig. 2); Dr. McDade retested serum from two patients of the Pennsylvania respiratory outbreak against what was cultivated in the yolk-sac culture and demonstrated reactivity via an immunofluorescent assay. CDC virologist Gary Noble then challenged Dr. McDade blindly with a panel of patient sera from both Pennsylvania patients as well as those with respiratory illnesses (including influenza). All of the Pennsylvania serum specimens reacted with the recovered yolk sac bacterium, with findings being so obvious that they were corroborated by an entomologist who happened to be working late that evening. Dr. McDade scribed the following perspective, “in this case, it was the ‘contaminant’ that needed to be isolated and identified, which seemed counterintuitive. And then, of course, when I eventually considered that possibility, I chastised myself for ‘daring’ to assume a bacterial etiology when all of the bacteriology laboratories at CDC had already eliminated a bacterial etiology.” Ultimately, he drew a rather service-oriented conclusion to this rather significant finding, “we were on our way. I’d like to say that at that point, ‘I had done my job because all that the CDC needed at that point was an isolate.’”

Dr. McDade remained connected to this investigation for another year. While cultivation of the disease agent directly from lung tissue was not realized at the time, the bacteriology laboratories at CDC were eventually able to accomplish *in vitro* propagation of the Legionnaires’ disease bacterium on Mueller Hinton agar supplemented with 2% IsoVitaleX (Baltimore Biological Laboratories, Cockeysville, Maryland) after prolonged incubation only when provided with yolk-sac emulsions (again, derived from diseased experimental guinea pigs). This finding left Dr. McDade to opine, “one of the things that I wish I would have been able to follow up on was ‘was there something that was carried over?,’ ‘was it something from the egg?,’ ‘was the Legionnaires’ disease bacterium (if it grows in living tissue) producing something that it doesn’t make on agar?’ To this day, I wonder whether or not the isolates that they got on inanimate media truly reflect everything that the organism can produce.” It is not unreasonable to speculate that this potential *in vivo* and *in vitro* dichotomy, as well as subsequent work elucidating the L-cysteine growth requirement for the Legionnaires’ disease bacterium, was contributory to the extended time necessary to ascribe an etiology to this summer 1976 respiratory disease outbreak. (This topic will be revisited later in this feature.)

At the 1977 American Society for Microbiology General Meeting in New Orleans, Louisiana, Dr. McDade presented findings on the Legionnaires’ disease bacterium and was queried by F. Marilyn Bozeman, a rickettsiologist from the United States Food and Drug Administration Bureau of Biologics. She suggested that the organism described by Dr. McDade reminded her of rickettsia-like agents that were previously isolated in guinea pigs by laboratories in the Washington D.C. area from a patient blood specimen in 1947 (15; isolate historically known as the OLDA strain) and lung tissue from a deceased individual with “skin diver’s disease” in 1959 (16; isolate historically known as the WIGA strain). Specimens were collaboratively shared and compared to organisms derived from the 1976 outbreak. It was reported that the Legionnaires’ disease bacterium and the OLDA strain comprised the same species (17). Both grew on charcoal-yeast extract agar, on Feeley-Gorman agar, and on enriched Mueller-Hinton agar, but not on blood agar, trypticase soy agar, or in thioglycolate broth. Both the Legionnaires’ disease bacterium and the OLDA strain revealed 39% G + C content and displayed reactivity with sera collected from patients involved in the Legionnaires’ disease outbreak. The WIGA strain was demonstrated to be antigenically similar to the Legionnaires’ disease bacterium, but yielded G + C content of 43%. At least one additional outbreak scenario, ironically in a Washington, D.C. hospital, was retrospectively linked to the Legionnaires’ disease bacterium in late 1970s literature (18). Dr. McDade and colleagues demonstrated 4-fold or greater increases in acute versus convalescent serologic reactivity (with minimum convalescent titer ≥64) to the bacterium in 85% of banked paired serum specimens collected from patients in the 1965 outbreak. Dr. McDade summarized the serendipity of all of this work, “this was one that had been around a long time; it was just a non-cultivable organism that fell through the cracks and it happened to come out just because somebody happened to have a rickettsiologist on staff at that moment who followed a protocol that probably began with Louis Pasteur…but it worked. I just felt like the right person at the right time.”

While these findings were landmark in nature, dissemination and (especially) publication of such findings became a source of self-scrutiny for Dr. McDade (as mentioned earlier) on the basis of the length of time necessary to determine the source of the outbreak. One subsequent publication that may provide further insight into the difficulty surrounding the identification of disease etiology (19) was written by Dr. McDade and his CDC colleague and mentor Dr. Charles Shepard, Chief of the Leprosy and Rickettsia Branch. The Legionnaires’ disease bacterium was cultivated from agar media, yolk sacs, or spleens of infected guinea pigs. After adjusting organism concentration, naive guinea pigs and yolk sacs were challenged with a standardized inoculum of the three sources of bacterium for subsequent determination of 50% lethal doses for these two hosts (YSLD_50_ for yolk sacs; GPLD_50_ for guinea pigs). The number of Legionnaires’ disease bacteria derived from yolk sacs and guinea pig emulsions that generated YSLD_50_ was approximately 6 log_10_ less than that required from cells cultivated *in vitro*. Bacterial load necessary to generate GPLD_50_ was approximately 4 log_10_ greater from inoculum propagated on agar medium versus that derived from yolk sac incubation. The authors concluded that a “virulent to avirulent” *in vivo* to *in vitro* conversion compromises organism recovery in a laboratory setting.

Dr. McDade described a very poignant conversation that followed. He wrote, “Shepard was very much a hands-off supervisor; during our time working on the Legionnaires’ disease bacterium, we would occasionally sit together and lay out the (few) joint projects we did, and then he would wait for me to come to him when I thought his input was needed. We met in his small office…to discuss the draft article. Shepard was by nature a very calm, deliberate person; I am a bit more excitable. So, after a while, when several sticking points remained, our conversation became a bit more ‘spirited.’ Finally, Shepard abruptly stopped talking. He put his hands together behind his head, looked up, and stared at the ceiling. His reading glasses rested on the tip of his nose. Neither of us said a single word for a full one to two minutes, which seemed like a very, very, long time. Finally, he turned to me and said, ‘you know, nothing that you write, and nothing that I say, can change the truth. The truth just is! It’s our job as scientists to find the truth the best way we know how.’ I had only been at the CDC for a relatively short time, but for the first time I realized that I had been formally introduced to the CDC ethos that was all around me…I like to think that the same ethos exists today.”

Pursuant to the aforementioned presentation of Dr. Huxsoll on tropical canine pancytopenia in the late 1960s, Dr. McDade stepped away from work on the Legionnaires’ disease bacterium and returned to the rickettsiology division in the early 1980s. He recalled receiving an “inquiry with conviction” in 1986 from Dr. Koichi Maeda, a pathologist from Henry Ford Hospital in Detroit, Michigan, regarding a potential Rocky Mountain Spotted Fever case, but “without spots.” Upon receipt of blood photomicrographs from Dr. Maeda, Dr. McDade immediately recalled the image from the presentation of Dr. Huxsoll. Subsequent studies of patient sera against known rickettsial antigen collections revealed the first reported case of ehrlichiosis in humans caused by *E. canis* (20). Dr. McDade summarized, “it turns out that there are niches in various parts of the United States with certain ticks where the organism does circulate. It’s funny—if you keep your eyes and ears open, sooner or later some of this dovetails. I think that I loved that discovery every bit as much as I loved the Legionnaires’ disease bacterium. It was being at the right place at the right time.”

As his career progressed, Dr. McDade stepped into bureaucratic roles in the CDC, first as Chief of the Viral and Rickettsial Zoonoses Branch from 1984 to 1988, then succeeding the retired Dr. Albert Balows (21) as Associate Director for Laboratory Science from 1989 to 1998. Ironically, Dr. Balows published a brief editorial in *Annals of Internal Medicine* (22), concomitant to the valid and effective publication of *Legionella pneumophila* (23), that encouraged providers and scientists alike to use the taxonomic classifications of pathogens such as *L. pneumophila* and *Legionella bozemanii* sp. nov (24). (now *Legionella bozemanae*) for communication rather than customary monikers such as “Legionnaires’ disease bacterium” and “Bozeman’s rickettsia-like bacterium,” respectively. In a further twist of fate, one can also appreciate the irony that the *Legionella* (ultimately elucidated by rickettsiologists like Dr. McDade) and *Coxiella* (the aforementioned Q fever ricksettsia) genera share taxonomic hierarchy within Order Legionellales—perhaps on the basis of their unique means of promoting intracellular survival (25, 26). Dr. McDade recalled that he enjoyed these CDC leadership opportunities. “Some of the things that I was able to do were very rewarding. Sometimes, there was a problem, and it was my job to fix it somehow, and I would learn from that. They weren’t always fun, but they were learning experiences.” Overarching among all of these remembrances was Dr. McDade’s dedication to CDC and its mission relative to public health. “I absolutely loved CDC—almost from the moment that I got there. The more that I became involved with various activities, the more I said, ‘this is where I belong.’”

In a later CDC leadership role, Dr. McDade served as Deputy Director of the National Center for Infectious Diseases from 1998 to 2001. A few years previous to that time, a seminal report entitled *Emerging Infections: Microbial Threats to Health in the United States* was released by the Institute of Medicine Committee on Emerging Microbial Threats to Health (27). When considering the content of that report, a rather holistic goal of Dr. McDade within his public health service realm became “trying to get people to start thinking about things in the broad context; and as a result of that, I think that they would have a newer appreciation of infectious diseases—such that involvement is not solely related to the pathogen. There are so many additional factors that could be involved…we live in a world; we’re not isolated in air-conditioned rooms and never have to go out.” Such thoughts and goals were the basis for his founding of the journal *Emerging Infectious Diseases*, with the first issue published in January of 1995. In conversation, Dr. McDade highlighted one paper on chytrid amphibian disease (28) (publication of which was criticized by some colleagues) that particularly subscribed to this isolationist philosophy. Chytridiomycosis is a highly transmissible fungal disease in frogs and toads that is caused by *Batrachochytrium dendrobatidis*. “I could see what it was doing. If it infects the amphibians, depending on where they were or when it would happen, fewer amphibians would be able to eat mosquitoes, and this would result in an increase in mosquito-borne diseases.”

Founding a peer-reviewed journal saw its set of challenges. “It wasn’t all that easy. We didn’t have a budget, but we did have a mailing list because there were a lot of things that went out from the CDC (such as *Morbidity and Mortality Weekly Report*), so we had a captive audience. We did have a lot of buy-in from people internal to the CDC, and they volunteered their time. To get it started, I had to solicit articles from a lot of people and hope that I identified people who would put something out that other people would read.” Trusted and knowledgeable editorial board members were sought by Dr. McDade for work on the quarterly-published journal. An additional barrier was grounded in the Science Citation Index, with a journal (at the time) having to be in existence for 5 years before published articles could be indexed. A negotiation to reduce this timeframe to 2 years allowed for an increase in quality articles from reputable sources during the infancy of the Journal. Dr. McDade transitioned to Orise Fellow and Ombudsman positions at the CDC from 2001 to 2005 and 2006 to 2007, respectively, and continued to consult on matters relative to *Emerging Infectious Diseases* (now published monthly and in its 32nd year of existence).

As one peruses the *curriculum vitae* of Dr. McDade, it is easy to note the approximately 100 book chapters, peer-reviewed primary publications, and manuals; editorial activities in venues such as *Emerging Infectious Diseases* and *Manual of Clinical Microbiology*; dozens of national and international presentations; distinguished military service; leadership in the American Society for Rickettsiology and Rickettsial Diseases (president from 1985 to 1986); awards such as the Secretary’s Award for Exceptional Achievement from the U.S. Department of Health and Human Services (1978), the Richard and Hinda Rosenthal Award from the American College of Physicians (1979); Docteur Honoris Causa from the University of Marseille, France (1991), the CDC William C. Watson, Jr. Award (1991), the Charles C. Shepard Lifetime Scientific Achievement Award (2001), and the Gen-Probe Joseph Award (2011); and, of course, directly being the subject of a bacterial taxonomy exercise (29). The career of Dr. Joseph E. McDade should also be celebrated for persistence, service, courage, and the ability to apply basic scientific talents in unusual and unexpected circumstances for the benefit of many.
